# "There Is No One Truth": A Qualitative Analysis of Advisory Committees in COVID-19 Policy-Making in Germany

**DOI:** 10.34172/ijhpm.9424

**Published:** 2026-08-01

**Authors:** Elisa Braun, Kerstin Sell, Evelien de Hoop, Karin Geffert

**Affiliations:** ^1^Institute for Medical Information Processing, Biometry and Epidemiology, LMU Munich, Munich, Germany.; ^2^Pettenkofer School of Public Health, Munich, Germany.; ^3^Athena Institute, Science Faculty, Vrije Universiteit Amsterdam, The Netherlands.

**Keywords:** Public Health, Evidence-Based Policy, COVID-19, Germany, Advisory Committees, Evidence Use

## Abstract

**Background:**

The COVID-19 pandemic posed an unprecedented public health challenge, causing over one hundred thousand deaths in Germany alone. As elsewhere, policy-makers in Germany sought expert advice to navigate uncertainty and urgency, including through ad hoc advisory committees. We aimed to identify committees operating between 2021-2023, assess their composition, and analyse their work processes with a particular focus on structures, expert deliberations and evidence use.

**Methods:**

We conducted a systematic document analysis of government documents and semi-structured interviews with committee members in April/May 2024. We identified documents (parliamentary inquiries and freedom of information requests) through systematic searches in digital government archives; and purposively sampled experts from identified committee member lists. Documents were analysed using abridged content analysis, and interview transcripts were analysed using codebook thematic analysis.

**Results:**

We screened 984 documents, analysed 43 documents, and conducted 12 interviews. Ultimately, we identified six ad hoc advisory committees which met our inclusion criteria. These committees advised the German national government and the state governments of Bavaria, North Rhine-Westphalia, Rhineland-Palatinate, Schleswig-Holstein, and Thuringia. These were dominated by academic experts, especially from bio-medical disciplines. *Structure of the advisory process:* Consultations were direct (in person/online meeting) or indirect (written reports). Direct consultations typically included short presentations/input, followed by discussions to reach a consensus opinion. *Expert deliberations drew* on expert opinion, reports, pre-prints, and peer-reviewed studies. Multi-disciplinary exchange was valued but faced time constraints; opinions differed on whether a successful multi-disciplinary exchange was achieved. *Reflections:* Committee members were aware of the limits of their advisory role but occasionally struggled with the political nature of the work.

**Conclusion:**

German policy-makers continued to involve ad hoc advisory committees after the first year of the pandemic. Where it was achieved and meaningful, multidisciplinary work was perceived as valuable. Political decisions deviating from expert advice required justification under certain circumstances.

## Background

**Key Messages**

**Implications for policy makers**

- Ad hoc advisory committees were set up in Germany during the COVID-19 pandemic to provide decision-makers with timely and accessible expert insights.
- These committees were dominated by bio-medical experts; some committees met directly with policy-makers while others communicated their advice through reports.
- In future crises, advisory committees should include social sciences expertise and the voice of affected groups to improve appropriateness and relevance of the advice.
- Direct interaction between policy-makers and advisory committees is also likely to improve the relevance of the advice and will enhance advisors’ understanding of policy-making.

**Implications for the public**

During the COVID-19 pandemic, many governments set up expert advisory committees to help make decisions in a highly uncertain situation. This study examined committees set up to advise the German national and state governments. We examined who served on these committees and how they worked together. Experts shared opinions, reports, and studies to inform discussions. While input from different fields was valued, time pressure and dominant voices sometimes made collaboration difficult. Members saw themselves as advisors, not decision-makers, but found it difficult to separate science from politics. In future crises, policy-makers should consider involving experts from social sciences to broaden the committees’ expertise. Representation of women should be ensured. Involving groups who are particularly affected by the crisis can make recommendations more appropriate and feasible.

The COVID-19 pandemic presented itself as a multifaceted public health threat causing more than 21 800 excess deaths between 2020-2023 in Germany.^1,2^ The pandemic caused by the infectious agent SARS-CoV-2 posed an unprecedented problem and led to significant social disruption in various sectors. Governing this complex issue posed a challenge for policy-makers who were responsible for creating effective yet balanced policies,^3-5^ which account for the different and sometimes conflicting priorities and circumstances of different population groups.^6^

 The urgency, uncertain trajectory, and initial lack of information regarding the pandemic added to this policy-making challenge.^5^ Policy-makers were faced with ambiguous, conflicting and changing evidence whilst aiming to develop appropriate policies.^3,5,7^ Globally, many policy-makers turned to expert advice to navigate this novel situation,^8-10^ including in Germany.^11^

 Such advice must be of high quality and provide timely, accessible expert insight from multiple disciplines and follow good practices.^12,13^ These include expert advisors providing evidence, knowledge and expertise to propose and evaluate solutions to pressing issues and draw the attention of politicians to unrecognised problems.^14-16^ In turn, following such advice based on research and evidence legitimises and justifies new policies. Evidence-based public health (EbPH) refers to decision-making based on a systematic and conscious integration of the best available scientific knowledge, the expertise of relevant experts and stakeholders and the affected population’s perspectives, values, and preferences in the context of health policy.^17,18^ However, policy-making rarely constitutes such a rational process and scientific knowledge is neither the only nor the most important basis of decision-making.^7,19,20^ In practice, decision-making in public health is often the result of social and political negotiation processes; decisions *should* be made based on scientific evidence, but – in reality – are not exclusively based on it.^21^ To recognise this, the term evidence-*informed* (as opposed to evidence-*based*) decision-making has been coined.

 Nonetheless, many countries have established structures and processes that promote using scientific knowledge in policy formulation.^9,10^ Advisory committees are one such venue and have been increasingly established in recent years – which is likely due to the increased complexity of political issues where an understanding of the scientific basis of the respective sector is important.^22^ This “scientization of policy-making”^23^ through expert advisory bodies has most commonly been established in the health sector, among others.^22^ It has been argued that governments aim to increase their policy legitimacy through advisory committees.^24^

*“Ad hoc” *advisory committees address a particular novel issue and are usually formalised networks of experts.^25^ They are short-term and usually dissolved when the issue is resolved.^7,16,26,27^ Such committees aim to provide knowledge, information, guidance, and advice to governments based on expertise and the best available evidence identifying options and strategies to solve the problem at hand.^5,16,28-31^ Their members are usually purposefully selected experts with an in-depth and wide range of knowledge, skills, and experience in a particular subject.^5,16,29^ They can operate outside rigid policy frameworks and have a flexible organisational structure.^26,32-34^

 It is unclear whether these characteristics and standard practices of expert committees were set up in similar ways over the course of the COVID-19 pandemic. Firstly, advisory committees established early in the COVID-19 pandemic—including the ad hoc advisory committees installed in Germany—included a much narrower range of expertise, involving mainly medical practitioners and scientific experts of epidemiology, virology, and infectious diseases.^11,13,35^ Existing literature on policy-making at times of crisis cautions that this narrow range of expertise can come at the expense of alternative viewpoints and knowledge.^12,36,37^ Secondly, based on experiences of previous public health threats, it is likely that the novelty of the disease, the urgency to act, and the respective uncertainty led to improvised advisory processes.^13^ For example, deliberations weighing different suitable measures and alternatives may have been cut short out of fear of delays.^38,39^ Furthermore, decisions might not always be evidence-based, even when they were labelled as such.^15,40^ The practices of ad hoc advisory committees installed in Germany during the COVID-19 pandemic were not transparently shared and are therefore currently not well-understood.^11^

## Objectives

 The aim of this study was to identify ad hoc expert advisory committees operating between 2021-2023 in Germany, to describe their composition, and examine their work processes, with a particular focus on structures, expert deliberations and evidence use in the COVID-19 pandemic.

## Methods

 We conducted (*i*) a systematic search for official government documents and (*ii*) semi-structured interviews; and undertook qualitative content analysis of documents and codebook thematic analysis of interview transcripts. We drew on the consolidated criteria for reporting qualitative research (COREQ)^41^ (Supplementary file 1).

###  Conceptual Framework

 We chose a framework of expert advice for policy-makers suggested by the Organization for Economic Co-operation and Development (OECD) to inform this work.^34^ The framework delineates four phases: *formulating the question*, *selecting the advisors*, *producing the advice*, and *communicating the advice *(Figure 1).

**Figure 1:**
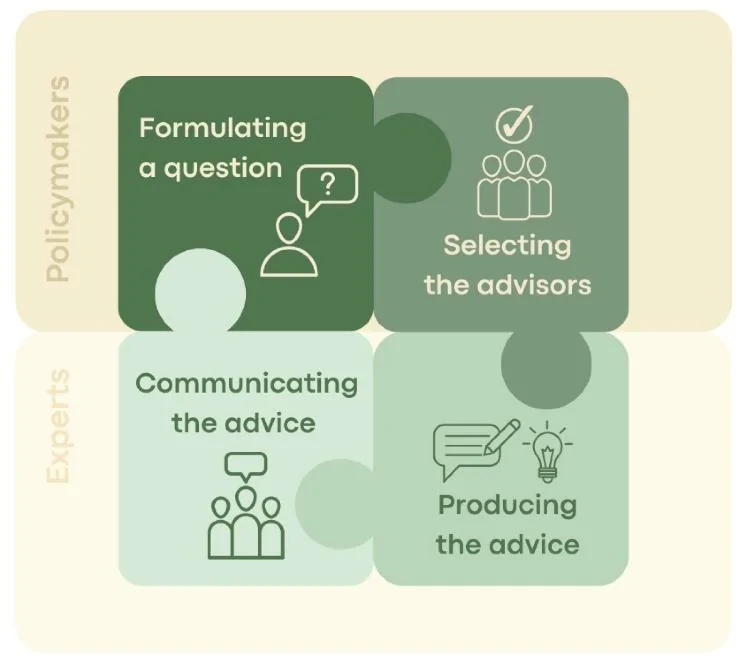
Advisory Process, Adapted From OECD, 2015. Puzzle pieces represent different phases that policy-makers or experts execute. Abbreviation: OECD, Organization for Economic Co-operation and Development.

 For *formulating the question*, the framework suggests that the end users of the advice should be involved. Secondly, adequate *advisors must be selected* to avoid conflicts of interest and secure the quality and legitimacy of the advisory process. The experts should have defined tasks and work independently from political input to *produce advice*. Scientific uncertainties should be evaluated and discussed. Lastly, the *advice is communicated* to the policy-makers, who subsequently can use it for new policy.^34^ During the whole process, transparency is of utmost importance. In a democratic system, where unelected individuals can influence policy-making and laws that affect people’s lives, it is good practice to disclose the sources of such influence to legitimize decisions, foster public trust, and enhance compliance. The recommendations resulting from expert advice and consulted evidence should thus be made publicly accessible; and policy-makers should explain their use of scientific evidence in decision-making transparently.^34^ In this study, we focussed on the phases of *producing *and *communicating the advice*.

###  Context

 Germany is a federal parliamentary republic made up of 16 federal states (Bundesländer). The federal government and state governments share power on health and health-related policy. The states are responsible for crisis management, including health emergencies, whereas the federal state is responsible for civil protection (eg, in times of war) and supports the states’ crisis management.^42^ To support this effort, Germany’s main public health agency, the Robert-Koch-Institut, hosts a national Expert Committee for Pandemic Respiratory Disease as a platform to complement the agency’s expertise.^43^ This expert committee supersedes the institution’s former Advisory Board on Influenza, which had been established as a key learning from the H1N1 pandemic in 2012.^43^

###  Document Analysis

####  Data Collection

 We followed an approach for the identification of documents undertaken in a previous study.^11^ We searched for minor inquiries^[1]^,^44^ and freedom of information requests^[2]^,^45^ to state and national governments, issued between 2021 and the end of 2023. This time frame was chosen to include documents not already assessed in a previous study.^11^ The end of 2023 marks the time the last COVID-19 measures were lifted.^46^ Minor inquiries were accessed through the digital document archives of the 16 state governments and the website of the national government of Germany. We trialled search terms for these databases and opted for narrow search terms (“COVID-19” and “Expert*”) due to technical limitations of the search function of the databases; and to limit the overall number of results. Freedom of information requests were obtained through the website *fragdenstaat.de* again using the simple search prompt “*Expertenräte corona”* (English: advisory committees COVID) due to technical limitations. We identified one document through interviews. Because our goal was to identify experts and expert committees, we did not pursue a comprehensive document review; instead, we deemed the identification of an anchor document sufficient.

 Search results were screened for relevant titles by one author (EB). Subsequently, the same author screened the full-text of documents to assess whether they reported on an advisory committee. We considered advisory committees as ad hoc and included the respective documents when they met previously suggested criteria^7,16,26-28^:

- a group of experts;
- appointed by policy-makers in Germany;
- which provided holistic advice on the COVID-19 pandemic to policy-makers; and
- which was installed as reaction to the pandemic and dissolved when no longer viewed as necessary.

####  Data Analysis 

 Our qualitative content analysis followed O’Leary’s procedure for public record documents.^47^ We conducted a pragmatic qualitative content analysis of documents to examine advisory committee composition. One author (EB) extracted information on committee members’ names and professional backgrounds.

 All committee members were individually categorised according to their disciplinary background (bio-medical vs other disciplines), current position (in particular if they were acting as medical practitioners), researcher (in terms of association with a research institution), and gender as deduced from the name or title. Occasionally, additional information about the individuals was retrieved from their institutional webpages. For the category *bio-medical* the following disciplines were included: hygiene, immunology, infectious diseases, medical practitioners, microbiology, molecular biology, pharmacology, vaccinology, and virology. Committee diversity was calculated by number of different disciplines divided by the number of advisory committee members.

###  Expert Interviews

 We conducted semi-structured interviews with members of ad hoc advisory committees. Semi-structured interviews are suitable for deep exploration of individual experiences and perspectives, providing rich, qualitative data that complements the document analysis. Furthermore, this interview format allows participants to explain their answers, giving context.^48^

####  Sampling

 Participants were purposively sampled from the list of committee members identified in the document analysis. We excluded committee members who were politicians, other representatives with executive power, church representatives and committee members without available email addresses. The purposive sample aimed at representation of the different states and disciplines (ie, medicine, natural sciences, sociology, economy, and psychology). The participants were contacted in three rounds. Firstly, to minimize recall bias, committee members who were active in 2022 or 2023 were contacted. Then, for non-participants, comparable substitutes were contacted in the second round. Finally, further experts were contacted in regions with fewer than two confirmed interviews. Every expert was approached through an invitation email with an information sheet and consent form and received up to three reminders. The recruiting period was from April 27, 2024 until May 31, 2024. We offered no financial compensation or other incentives for participating in the study.

####  Data Collection 

 Interviews were conducted in the videoconferencing software *Zoom* or via phone, depending on the interviewee’s preference. Interviews were conducted in German by one author (EB) and lasted between 28 and 57 minutes with an average of 48 minutes to accommodate the experts’ schedules. Following the interviewees’ consent, the interviews were recorded using the videoconferencing software’s recording function or a digital recording device. Field notes were taken by the interviewer during and after the interviews.

 Our semi-structured interview guide contained open-ended, deepening and prompt questions (Supplementary file 2). The open-ended questions encouraged participants to express themselves freely and allowed for adaptation based on the conversation flow and the exploration of new, exciting and unexpected themes. Thus, not every participant was asked the same questions, and therefore, not every participant voiced their perspective on all topics. To maintain comparability between interviews, the three main topics of the interview guide—committee meeting characteristics, expert deliberations and communicating the results of the counselling processes—were, however, always discussed.

 All questions in the first version of the interview guide were derived from the conceptual framework and were subsequently adapted in an iterative manner. Adaptations were made when multiple participants brought up the same topic that was not previously included in the interview guide.

####  Data Management

 All data collected were stored on the secure institutional information technology infrastructure provided by the Institute for Medical Information Processing, Biometry, and Epidemiology (IBE) at the Ludwig-Maximilian-University (LMU) Munich. The IBE independently operates a complete information technology infrastructure in its own protected server rooms, which meets the requirements of an institute for research on highly sensitive patient and study data. Within this infrastructure, a separate isolated storage area was provided for the project. Raw data was only accessed by one of the main researchers (EB) and the supervisor (KG). However, the number key was solely accessible through a password by the main researcher.

 Participants were informed about the handling of their data by means of an information sheet. This follows the recommendations of the Ethics Committee of the Medical Faculty of the LMU.

####  Data Analysis 

 We transcribed interview recordings using the *noScribe* software,^49^ which is artificial intelligence (AI)-based, free of charge, and open-sourced. The software runs entirely offline and processes both audio and transcripts locally on the user’s device. This ensures that no personal data is transmitted to external servers or cloud-based AI services. As *noScribe* does not collect or share data, it aligns well with data protection standards and was approved by the ethics committee. The transcripts were compared with the interview recordings, pseudonymised, and subsequently imported into the qualitative analysis software MAXQDA 24.^50^ We analysed transcripts in German language and the lead author (EB) translated illustrative quotes to English for this manuscript. The accuracy of the selection and translation of the quotes was reviewed by the senior author (KG). We conducted thematic analysis from a realist perspective which focuses predominantly on explicit (*semantic*) rather than implicit (*latent*) codes^51,52^ and used a codebook thematic analysis approach.^53^ The lead author (EB) drew on relevant theory to deductively develop an initial set of codes, which were collated in a code book.^54^ Afterward, the researcher familiarised herself with the data. The researcher (EB) inductively added additional codes that covered aspects that were prominent in the interviews. Double coding of four transcripts was performed by the lead author (EB) and senior author (KG) to enhance the quality of the data analysis.^55^ More codes were added after additional in-depth vertical analysis of the data. Then, the code book was refined by merging codes into overarching themes and checking if the overarching themes depicted the data by a horizontal analysis of all data (Supplementary file 3). Lastly, each theme was defined and described, with themes being demarcated from one another before producing the final report. In our write-up, we indicate how many interviewees commented on a specific theme.

 During data analysis, one interview was excluded from the sample as the interview participant was not part of an ad hoc advisory committee as described in the eligibility criteria.

## Results

###  Advisory Committee Composition

 Overall, we screened 18 websites, assessed 984 documents for eligibility, of which we included 43 (Supplementary file 4). Ultimately, we identified six ad hoc advisory committees. One committee advised the national government. The remaining five committees advised the state governments of Bavaria, North Rhine-Westphalia, Rhineland-Palatinate, Schleswig-Holstein, and Thuringia, respectively.

 The numbers of advisory committee members ranged from 19 (national government) to six (Bavaria) and members were mostly affiliated with universities, research institutions, or worked as medical practitioners (Table). The Thuringian committee included the most disciplines in absolute numbers. The committee of the national government had the highest percentage of bio-medical experts, while North Rhine-Westphalia had the lowest (Table). Women made up between 0% (Rhineland-Palatinate) and 46% (Schleswig-Holstein) of committee members (Table).

**Table:** Overview of Ad Hoc Advisory Committees Which Advised German Governments in the COVID-19 Pandemic (2021-2023)

**Region**	**Committee Name (Period of Activity)**	**Number of Members**	**% MP or Researcher**	**Number of Disciplines**	**Disciplinary Diversity**^a^	**% Bio-medical**	**% Women**	**Number of Study Participants**
National government	Expert committee of the German Government 2022^b^ (12/2021-04/2023)	19	94%	10	53%	68%	32%	3
Bavaria	Round Table Bavaria^c^ (12/2020-02/2021)	14	93%	10	71%	36%	14%	1
North Rhine-Wesphalia	Expert committee^d^ (04/2020-06/2021)	12	75%	9	75%	8%	42%	0
Rhineland-Palatine	Constant Advisors^e^ (NA)	7	71%	6	86%	71%	0%	0
Schleswig-Holstein	Expert committee^f^ (04/2020-02/2022)	10	80%	8	80%	50%	40%	2
Thuringia	Scientific committee for SARS-CoV-2^g^ (06/2020-06/2021)	13	92%	11	85%	15%	46%	5

Abbreviations: MP, medical practitioner; NA, not available. ^a^ Disciplinary diversity = the degree of diversity with 100% being the maximum diversity, where each member represented a different discipline that no other member represented. Disciplinary diversity was calculated by dividing the number of committee members by the number of disciplines. Original names in German: ^b^ Expertengremium der Bundesregierung 2022; ^c^ Runder Tisch Bayern, period of activity refers to official data reporting about the meetings; ^d^ Expertengremium; ^e^ Ständige Berater; ^f^ Expertengremium, period of activity refers to the first and the last meeting that were identified via official documents; ^g^ Wissenschaftlicher Beirat zu SARS-CoV-2.

###  Advisory Committee Process

 Of 69 eligible committee members identified in documents, we contacted 60 individuals, of which twelve agreed to participate in interviews (response rate 20%; Supplementary file 5). Representatives of the national government, Bavaria, Schleswig-Holstein and Thuringia advisory committees agreed to participate, representing 16%, 7%, 20%, and 38% of members of their respective committees (Table).

 In interviews, we identified three key themes: the *structure of the advisory process*, *expert deliberations* within the advisory process and *reflections on the advisory process* (Figure 2).

**Figure 2:**
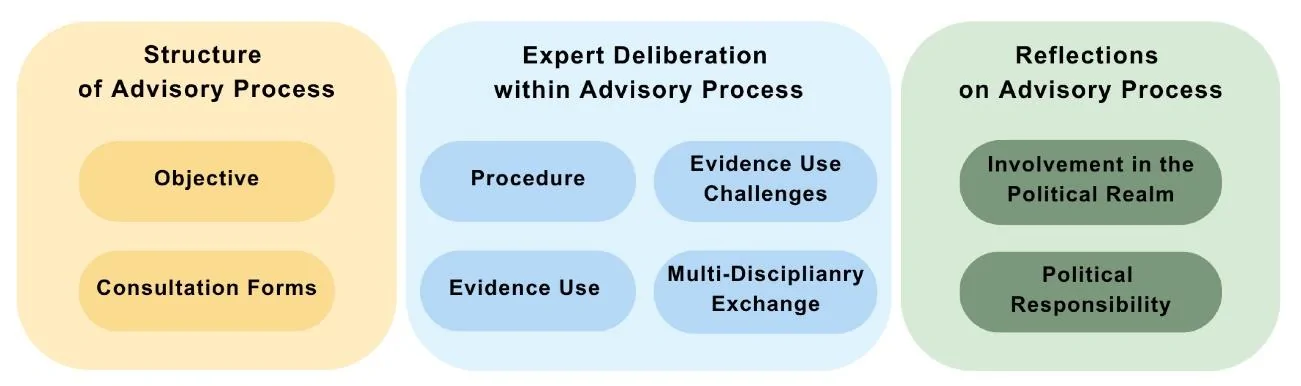
Overview of Main Themes and Subthemes Identified Through Codebook Thematic Analysis of Interview Data.

###  Structure

####  Objectives

 The overarching objectives of the advisory committees appeared uniform: Political decisions taken in the context of pandemic response – like the implementation of public health measures – should be informed through scientific expert advice (n = 9). Therefore, the advisory committees assessed the pandemic situation and responded to specific questions posed by politicians (n = 3). Bavarian and Thuringian committees specifically highlighted issues that had not been taken into consideration by the policy-makers (n = 5). The advice was usually given in the form of recommendations (n = 8). In contrast, the Schleswig-Holstein committee developed concrete proposals, which served as a direct basis for decision-making by the state cabinet (n = 1).

 When asked about the selection process for expert committees, interviewees were not aware of the reasons for their nomination.

####  Consultation Forms

 The committees advised policy-makers indirectly, through written advice, and directly at committee meetings or in formal or informal exchanges. Indirect consultations predominated in the Thuringian committee, which issued so-called *EULEN-Gremien* to transport their advice to the policy-makers (n = 4). *EULEN-Gremien* documented the extent to which scientific positions on given topics were controversial (n = 1). If the scientific position on a topic was unanimous, political decisions deviating from the evidence needed more substantial justification by the policy-makers, which was mentioned by one participant and supported by the official report of the committee.^56^ Direct consultationwith policy-makers in committee meetings, with simultaneous production and communication of advice, was additionally used by the Thuringian committee and predominated in the national government committee. In Thuringia, policy-makers were present for individual agenda items (n = 1), whilst at national government committee meetings policy-makers were continuously present, but not always the same representatives (n = 3). In both committees, this form was chosen when policy-makers sought direct advice on a specific topic (n = 6). In the national government committee, the policy-makers also gave input regarding the decision-making processes (n = 3). One interviewee pointed out that the presence of the policy-makers might have increased the likability bias of the experts which in turn may have influenced the deliberations (n = 1). Both committees issued written, publicly available statements to demonstrate their work to society, upholding transparency (n = 3).

 The meetings of the Schleswig-Holstein committee were led by the prime minister and sometimes the whole cabinet was present; in Bavaria, committee meetings were fixed appointments with the responsible ministry (n = 2). Both in Schleswig-Holstein and Bavaria, policy-makers were reported to have actively participated in the discussions; no written documentation was issued after such meetings (n = 3).

 Finally, direct exchanges with policy-makers also took place outside of planned committee meetings. In formal direct exchanges, representatives of the Thuringian committee attended meetings of the State Chancellery or work meetings of policy-makers (n = 3). In informal direct exchanges, representatives of the Bavarian committee interacted with policy-makers via personal contacts via telephone (n = 1). This included exchanges with the ministers, even on weekends or evenings (n = 1).

###  Expert Deliberation

####  Procedure

 In all advisory committees, expert deliberations involved expert input, discussion among committee members and aimed at reaching consensus statement. Meetings typically commenced with a short presentation from an expert (n = 2), expert input from various fields (n = 4), or expert statements (n = 1). In Schleswig-Holstein, this input was given by the prime minister. Afterwards, experts could contribute further insights based on their discipline (n = 1).

 “*For example, a new variant has emerged. Someone then presents the data that is available so far. Then you think what consequences does that have? What does it mean for Germany? Then the person doing the simulations says what the simulation model says. The infectiologist gives their assessment. The immunologist says what their assessment is. And then you try to find a consensus opinion” *(P02).

 Subsequently, arguments were carefully discussed, and it was debated which statements to agree on (n = 2). The ensuing discussions were described as sometimes involving controversial debate (n = 1).

 “*There is no one truth. It depends on which criterion you look at. Can children be prescribed a mask? You can [prescribe them to wear a mask]. Not really a problem from a medical point of view. Psychologically, perhaps more of a problem. Ethically, depending on which ethics you hold. It’s important to me to protect many people. Or to protect the rights of the individual. This does lead to discussion” *(P05).

 However, typically, a consensus statement emerged from these discussions, with almost all statements being unanimously adopted (n = 2).

####  Evidence Use

 Advisory committee deliberations drew on expert opinion, reports, pre-prints, and peer-reviewed studies. The latter only became available over the course of the pandemic and experts faced great uncertainty at the onset:

 “*The work of these advisory bodies is essential, but it is also crucial to understand that you are providing expert advice, yes, but with a lot of uncertainty, time pressure, and lack of information” *(P02).

 The form of input thus depended on the time period in the pandemic, available information, and the area of advice required. For example, studies examining the economic impact of the pandemic were conducted later than those investigating its impact on public health (n = 6). When peer-reviewed studies were not (yet) available mainly expert opinion informed the advice. Committee members understood this as an expert’s opinion based on their acquired knowledge through experience and training or rationality and logic of the discipline (n = 5). As long as the availability of evidence specific to the COVID-19 pandemic remained limited peer-reviewed studies on comparable past events provided the opportunity to draw analogies. This entailed, for example, consulting evidence on previous influenza and SARS outbreaks to assess possible progressions of the pandemic; but also previous teacher strikes and extended summer holidays to assess consequences of school closures as public health measures (n = 2).

 To obtain information on the current epidemiological situation, committee members drew on officially issued reports by the national public health agency Robert Koch-Institute (RKI) or the World Health Organization (WHO) (n = 2). In combination with clinicians’ observations from their work, reports provided information on patient numbers and status, death cases and the occupancy of clinical wards (n = 4). Additionally, preprints served as input for deliberations, which were mainly used by medical practitioners (n = 2).

 Peer-reviewed studies were used in expert deliberations as they became available (n = 1). Some committee members conducted literature searches and reviews of the available evidence – aiming to elicit the scientific consensus on a given topic (n = 5).

 Critically, participants noted that it was not always clear whether statements were based on personal experience or backed by peer-reviewed evidence.

####  Evidence Use Challenges

 Evidence use was constrained by inconclusive evidence, limited data access, and practical constraints. Many participants reported that the initial studies on the pandemic were often preliminary and sometimes inconclusive or even contradictory (n = 4), which left room for interpretation (n = 1). Other participants mentioned that personal preferences, attitudes and convictions likely influenced the deliberations, even if committee members tried to avoid this (n = 2), and which could not be entirely avoided given that there was no “one truth” (n = 1).

 Furthermore, the access to evidence was sometimes limited. Participants highlighted that the availability of Germany’s public data records was extremely poor compared to other countries (n = 2). This is largely due to Germany’s fragmented federal system, which, at the beginning of the pandemic, lacked a centralised recording system for tracking COVID-19 incidence, side effects of measures, or availability of intensive care beds (n = 1). Additionally, due to stringent data protection regulations in Germany, there was poor availability of medical data and the absence of registers for vaccinations, which hindered effective pandemic management and diminished reliable data for expert deliberations (n = 2). Efforts to improve data exchange systems were only established during the pandemic.

 Furthermore, committee members faced significant practical constraints in systematically identifying and analysing evidence due to time pressure and limited financial and human resources (n = 5). Initially, the need for quick decision-making exacerbated these constraints, though the situation improved slightly as the committees adapted to the pandemic (n = 1). Despite recognising the need for additional support (eg, research assistants) the committees lacked the necessary funding and staff, with only minimal support available for taking brief minutes during committee meetings (n = 3). This lack of resources hindered their ability to conduct in-depth analyses and comprehensive research even when studies and evidence were available (n = 1).

####  Multi-disciplinary Exchange

 The advisory committee members provided a multitude of disciplinary perspectives. This was viewed as both valuable and sometimes challenging.

 Committee members reported constructive and open discussions aiming at incorporating viewpoints from different disciplines. Thus, efforts were made to communicate comprehensibly to ensure that topics were understood across different disciplines (n = 2) encouraging experts to reconsider their positions (n = 2).

 “[*T]here are also situations where you might think that this is a purely virological question. Still, then, the economist discovers an aspect that needs to be taken into account from this perspective or the field of law or psychiatry” *(P01).

 Opinions differed on whether a successful multi-disciplinary exchange was indeed achieved. One participant enthusiastically said so:

 “*In this respect, we didn’t have a kind of ‘one person decides here’ phenomenon, but a very good culture of discussion and decision-making” *(P01).

 This included sessions in Thuringia attended by the Prime Minister, with discussions remaining open and participants feeling able to ask questions and contribute (n = 1). In the national government committee, another participant described the discussions as typical interdisciplinary scientific communication, with all members contributing openly and equally (n = 1). However, others noted that a few individuals dominated the conversation (n = 1) and that certain disciplines, such as medicine, law, and ethics, played a more central role than others (n = 1).

 Another participant acknowledged the benefits of interdisciplinary discussions to enrich the decision-making process but pointed out its disadvantage, namely slowing down the process (n = 1). This was seen as problematic given the urgency of the situation (n = 1), as echoed by others (n = 1). However, others reported that the efficiency improved over time as meetings became more structured (n = 2).

###  Reflections

####  Involvement in the Political Realm

 Committee members appreciated the role of the advisory committees whilst some struggled with the political nature of this work. Many participants explicitly mentioned having experienced the advisory process as a meaningful and positive experience (n = 5). However, some found it challenging to be involved in a political process. One participant mentioned that their committee was dissolved before they could finish their evaluations because another topic had become more relevant on the political agenda (n = 1). The same individual commented that they perceived politicians as using the advisory committees as a political tactic to *“perhaps [make] themselves look better”* (P05), ie, by framing unpopular measures as based on expert advice and framing popular measures as political decisions. Another committee member used more drastic words, describing the national government committee as a *“show commission […] to appease the public”* (P10). Whilst such an extreme opinion was not representative, committee members overall were well aware of the political nature of their work.

####  Political Responsibility

 Despite some frustration, when their advice was not implemented, experts recognised that their role was purely advisory and acknowledged that the decision-making power ultimately rested with elected representatives, who had to consider the broader political contexts, including social consensus and political responsibilities (n = 3).

 “*Ultimately, we act in an advisory capacity. You always must be aware of that. I am not a politician. I also don’t have to take responsibility for what I advise, like a politician […]. That’s why, in my view, it’s also up to the politician to deviate a little, as [they] then create [policies] at the end, where [they] think that there is perhaps more of a social consensus if [they] modify it in such a way. That’s always the case in consultation” *(P03).

 In this vein, committee members emphasised that they did not make specific recommendations beyond existing legal mandates (n = 2). The *EULEN-Gremium* set up in Thuringia was unique in this respect as it outlined scientific disagreements and required policy-makers to justify deviating from expert advice under certain circumstances.^56^

## Discussion

 This study identified six ad hoc advisory committees installed in Germany to advise policy-makers during the COVID-19 pandemic (2021-2023). The national and five state committees were dominated by academic experts, particularly from bio-medicine. Consultation forms were direct (in person/online meeting) or indirect (written reports). Typically, direct consultations included short presentations, followed by discussions to reach a consensus opinion on a given subject. Expert deliberation drew on expert opinion, reports, pre-prints, and peer-reviewed studies but it was usually not clear what type of evidence experts drew on. Multi-disciplinary exchange was valued but occasionally challenging due to time constraints and discursive dominance of certain disciplines. Committee members viewed their role as advisory with decision-making power resting with policy-makers but occasionally struggled with the political nature of the work. They navigated the latter in various ways. This included recognising that people with different perspectives experience different *truths*; and making scientific disagreements explicit to policy-makers.

###  Advisory Committee Composition

 We identified six ad hoc advisory committees with variable member composition which tended towards a predominance of experts from bio-medical disciplines. Women were under-represented. There was no data to assess underrepresentation in other potentially relevant categories, eg, ethnic background or place of residence. These findings align with other work. Previous work on advisory committees in Germany identified 21 advisory committees, of which information on committee members was available for 11.^11^ The difference in the number of advisory committees identified is mainly due to timing as we only considered committees active from 2021 onwards, whilst earlier work focused on 2020.^11^ The committees identified in the previous work^11^; and in international COVID-19 decision-making and expert task forces set up early in the pandemic were also dominated by men.^8^ Gender imbalance in the latter was particularly striking, with only 3.5% achieving gender parity and 82.5% mostly made up by men.^8^ Similarly, bio-medical expertise has dominated COVID-19 advisory processes.^11^ The lack of diversity with respect to gender and disciplines risks overlooking critical perspectives and expertise to address ethical, social, political, and ecological implications of the pandemic – including adverse unintended consequences. These are, however, key considerations when designing public health measures.^57,58^ Whilst the presence of much bio-medical expertise early in the pandemic may not be surprising, persistent dominance of these disciplines may have limited the capacity of the advisory committees to give meaningful advice on emergent pandemic challenges. The voices of non-experts including people with lived experience were entirely absent from the committees we identified, which is likely to have limited expert insights into feasibility and implementation of public health measures.

 With respect to the nomination of members, other work examining COVID-19 advisory committees in Italy, the United Kingdom (UK), United States (US), Uganda and Poland showed some similarities but also differences to our findings. In Italy, the US, and Uganda, committee members were assigned through political alignment (eg, party membership); in the UK and Poland, members were mainly from medical and public health disciplines, with few exceptions.^59^ The latter resonated more with our findings as we did not identify any nomination linked to party affiliation. In the same study, the authors concluded that policy-makers chose ad-hoc advisory committees over existing advisory mechanisms “to better control science and to steer it towards specific political objectives.”^59^ We did not investigate the underlying political rationale for implementing the advisory committees in depth but this conclusion corresponds to participants describing the advisory committees as *political tools.*

###  Advisory Committee Process

####  Structure 

 The uniform objective of the ad hoc advisory committees was to provide policy-makers with scientific advice, on topics defined by the latter. This predefinition of the role of the experts has been described by Pielke as the *science arbiter* role – one that responds to specific scientific questions raised by policy-makers.^60^

 While the objectives were comparable between the committees, the consultation forms differed. Interestingly, the phases of advice production and communication within the OECD framework were not always distinct phases in reality.^34^ Only in indirect consultations (Thuringia, Bavaria) policy-makers and advisory committees worked separately, with the advisory committee producing advice in internal discussions and communicating it in written format. Such closed-door discussions allow for high-quality expert deliberation, which can be viewed by the public as highly credible.^61,62^ However, this approach comes at the expense of the relevance of the advice; and limits advisors and policy-makers interaction and alignment of political priorities and the content of the advice. Indeed, for advice to be of high relevance, open discussions where producers and users of the advice deliberate collaboratively are considered more suitable due to their timely manner.^28,62,63^ This approach resembles the direct consultation forms predominantly described for the other committees. One rationale for this form of consultation mentioned by participants was that politicians, in part, could evaluate trade-offs between different values and goals with the experts and give their input concerning current political developments, in line with calls for “good governance of evidence.”^20^

####  Expert Deliberation 

 The expert deliberations were comparable between committees. Interviewees perceived the work among representatives of various disciplines as respectful, and enriching – whilst some participants noted time pressure as hampering this multi-disciplinary exchange and described medicine, law, ethics as dominating the discourse. This was not surprising as such multi-disciplinary work is difficult, especially considering communication and mutual understanding.^64,65^ Another limiting factor of multi-disciplinary work concerned committee size, with some interviewees claiming that large committee sizes made deliberations longer than necessary to reach a consensus, which they considered incompatible with the urgency to act in a public health crisis. This resonates with research on advisory committees working in various settings and jurisdictions, which demonstrated that small committees were more flexible but larger committees were more diverse.^22,65^

 Interviewees described both controversial discussion and subsequently reaching consensus. While our data do not enable us to explore the processes that facilitated reaching a consensus it appears likely that experts strove for consensus to reduce decision-making complexity at a time of crisis.

 According to EbPH principles, decisions on public health measures should be founded on relevant evidence and expertise, in conversation with local values and preferences.^21^ Interviewees reported that their deliberations incorporated scientific and practice-based evidence and drew on a broad spectrum of inputs ranging from expert opinion to peer-reviewed articles and reviews. The input type primarily depended on availability and the participant’s discipline. Examining a spectrum of evidence is a common phenomenon in multi-disciplinary work, as not all academics adhere to the same hierarchy of evidence. Thus, for example, Cairney adopts a broad evidence definition integrating quantitative and qualitative data.^66^ Also, Rutter and colleagues emphasise this broad definition, along with the importance of utilising a plurality of different methodological approaches.^67^ In our work, it remained unclear if, for example, qualitative research was considered, nor was it clear whether expert input or arguments were based on lived experience or evidence. The methods and data sources of evidence used in the advisory process should be explicit and transparent.^17^

 Advisory committee members operated amidst uncertainty, which is inherent in public health decision-making.^15,21,68,69^ Even when reliable scientific evidence is available, this evidence has limitations and will only produce simplified approximations of natural phenomena.^70^ Therefore, decision-making in public health should depend on reliable data analyses as well as sound (ethical) reasoning that ascribes value and normative judgment to empirical facts.^70^ Most committees considered the importance of such reasoning by including ethicists. Critically, we did not identify any meta-discussion of the available evidence and of the underlying, implicit norms shaping what different experts and disciplines consider *best available* scientific evidence in their respective fields. Thus, while in principle, the advisory committees aligned with two of the three pillars of EbPH – drawing on the best available scientific evidence as well as the experience and expertise of appropriate experts – the perspectives of interest groups were not represented; nor was the messiness of what constitutes evidence in a messy, uncertain situation reflected on.

####  Reflections

 Advisory committee members articulated that the responsibility for acting on expert recommendation rested with policy-makers who might or might not take on the advice, dependant on the result of complex, dynamic, societal, and political negotiations. This reflects the core principle of evidence-informed public health, namely that scientific evidence—in this case, in the form of expert advice—should support, not replace, democratic decision-making and societal negotiation processes.^71,72^

 Committee members took on the role of *experts facing political issues*, one of five self-images of researchers as identified in a qualitative study examining the science-policy interface in COVID-19 policy-making in Germany.^73^ In this role, collaboration with policy-makers is the norm, whereas the “scientific study supplier” is not likely to communicate research to evidence users; and the “public informer” will be more likely to address the general population.^73^ Two interview participants who described disillusionment with the advisory committees can be characterised as “restrained scholars” who withdraw from public or scientific discourse as they experienced limitations of expert advice.^73^ Advisory committees cannot perform all roles equally well as an increase in connectedness to decision-makers may come at the expense of the role of independent knowledge brokers.^22^ This resonates with the self-reflections of the interviewees on their role. From the role description of the interviewees, we conclude, however, that most of the interviewed experts understood their role as independent scientific experts with limited interaction with the political sphere.

 Similarities between Germany’s advisory committees and those examined in other research with respect to committee composition, nomination, and roles lead us to conclude that our work is generalizable to other settings, ie, countries with advanced systems for science-policy exchange and biomedical dominance of health discourses.

###  Strengths and Limitations

 This study has several strengths. Combining the document analysis and interviews revealed novel insights about the COVID-19 advisory committees in Germany. We conducted thorough and systematic searches for official government documents which gave us great confidence that we identified all advisory committees that were operating from 2021 onwards. The repeated purposive sampling allowed us to enrol a diverse set of committee members who were willing to discuss the work. Throughout the data analysis, we iteratively discussed themes to strengthen our findings. The double coding of four interviews further increased the rigor of the analysis.

 The study has the following limitations. Firstly, the included document types do not guarantee identification of all committees. We were not able to enrol members from two of the six advisory committees. Hence, conclusions from our study may or may not be generalisable to the other committees identified in our study and to committees elsewhere. However, eleven interviews can be considered sufficient as the aim was to discern themes concerning views and experiences among a relatively homogenous group.^74^ Recall bias was reported by participants as some of the events happened up to three years ago. Real-time observation of advisory committee meetings would have strengthened, in particular, our insights into how evidence was deliberated under uncertain circumstances. Due to the broad variety of topics, we were not able to explore all aspects in depth, for example considerations related to the quality appraisal of different types of data (eg, pre-prints, expert opinion) or aspects related to the transfer of international evidence and guidelines to the local level. Lastly, the interviews were conducted shortly after the publication of the RKI files, previously unpublished minutes from RKI meetings in the pandemic.^75^ This might have influenced participants to emphasise the positive aspects related to their work on ad hoc advisory committees.

## Conclusions

 The present study examined the composition and work processes of ad hoc advisory committees set up by German policy-makers during the COVID-19 pandemic and operating from 2021 onwards.

 Experts described direct and indirect interactions with policy-makers; with the former recommended to help tailor the advice and to improve experts’ understanding of the policy process. In the expert deliberations, multidisciplinary exchange was valued but it was curtailed by the dominance of biomedical experts and time pressure.

 It remained largely unclear how experts discussed the rigor and suitability of different types of evidence and how they negotiated differing epistemological positions. Attention to these questions in both research and in the implementation of advisory committees is warranted and may help strengthen genuine disciplinary plurality in the future.

## Acknowledgements

 The authors would like to thank Eva Rehfuess for her support of this work.

## Disclosure of artificial intelligence (AI) use

 Authors used noScribe software for the transcription of interviews and Grammarly software for the editing of the manuscript.

## Ethical issues

 The Ethics Committee of the Medical Faculty of the LMU has approved this work (project number: 24-0198).

## Conflicts of interest

 Authors declare that they have no conflicts of interest.

## Endnotes


^[1]^ Parliamentary groups or members of parliament in parliamentary groups can request written information from the Federal Government on specific issues by submitting a minor inquiry. They are mainly used by the opposition to inspect the government and obtain information and statements. A minor inquiry therefore entails the question of a member of parliament answered by the government.^44^
^[2]^ The Freedom of Information Act creates an unconditional right of access to official information and inspection of official files from federal authorities. The freedom of information requests therefore entail and an answer of the authorities.^45^

## Supplementary files


Supplementary file 1. Reporting Checklist.

Supplementary file 2. Interview Guide.

Supplementary file 3. Code Book.

Supplementary file 4. Flow Chart.

Supplementary file 5. Recruitment Process.
