# Supplementary material for: "There Is No One Truth": A Qualitative Analysis of Advisory Committees in COVID-19 Policy-Making in Germany
Source: Int J Health Policy Manag. 2026 Aug 1;15:9424. doi: 10.34172/ijhpm.9424 (PMC13770243; doi:10.34172/ijhpm.9424)
Supplement: Supplementary file 1 [file ijhpm-15-9424-s001.pdf]

Article title: "There Is No One Truth": A Qualitative Analysis of Advisory Committees in COVID-19 Policy-Making in Germany
Journal name: International Journal of Health Policy and Management (IJHPM)
Authors' information: Elisa Braun[1,2,3]¶, Kerstin Sell[1,2]¶, Evelien de Hoop[3], Karin Geffert[1,2]*
[1]Institute for Medical Information Processing, Biometry and Epidemiology, LMU Munich, Munich, Germany.
[2]Pettenkofer School of Public Health, Munich, Germany.
[3]Athena Institute, Science Faculty, Vrije Universiteit Amsterdam, The Netherlands.
***Correspondence to:** Karin Geffert; Email: kgeffert@ibe.uni-muenchen.de
Citation: Braun E, Sell K, de Hoop E, Geffert K. "There is no one truth": a qualitative analysis of advisory committees in COVID-19 policy-making in Germany. Int J Health Policy Manag. 2026;15:9424. doi:10.34172/ijhpm.9424
Supplementary file 1. Reporting Checklist

Table 1: COREQ Criteria. Consolidated criteria for reporting qualitative studies (COREQ): 32-item checklist from Tong (2007) DOI: 10.1093/intqhc/mzm042

Item	Question	Description
**Domain 1: Research team and reflexivity**		
**Personal Characteristics**		
**1. Interviewer/facilitator**	Which author/s conducted the interview or focus group?	Lead author
**2. Credentials**	What were the researcher's credentials?	B.Sc.
**3. Occupation**	What was their occupation at the time of the study?	M.Sc. Student
**4. Gender**	Was the researcher male or female?	Female
**5. Experience and training**	What experience or training did the researcher have?	B.Sc. in Molecular Medicine. Training in qualitative research though M.Sc. in Biomedical Sciences with specialization in Science in Society.
**Relationship with participants**		
**6. Relationship established**	Was a relationship established prior to study commencement?	There was no prior relationship between the main author and the participants of this study

**7. Participant knowledge of the interviewer**	What did the participants know about the researcher? e.*g. personal goals, reasons for doing the research*	Participants were informed about the aim of the study and the research question prior to the interviews, as well as the background of the first author
**8. Interviewer characteristics**	What characteristics were reported about the interviewer/facilitator? e.g. *Bias, assumptions, reasons and interests in the research topic*	Special interest in science in society, use of science to solve complex societal issue such as public health crisis, therefore focus on use of science in public health policy during COVID-19
**Domain 2: study design**		
**Theoretical framework**		
**9. Methodological orientation and Theory**	What methodological orientation was stated to underpin the study? *e.g. grounded theory, discourse analysis, ethnography, phenomenology, content analysis*	Thematic analysis from a realist perspective using a codebook thematic analysis approach
**Participant selection**		
**10. Sampling**	How were participants selected? *e.g. purposive, convenience, consecutive, snowball*	Purposive
**11. Method of approach**	How were participants approached? e.*g. face-to-face, telephone, mail, email*	e-Mail
**12. Sample size**	How many participants were in the study?	12
**13. Non-participation**	How many people refused to participate or dropped out? Reasons?	none
**Setting**		
**14. Setting of data collection**	Where was the data collected? e.*g. home, clinic, workplace*	Interviews were carried out via telephone and virtually, so it was the choice of the participants

**15. Presence of non-participants**	Was anyone else present besides the participants and researchers?	No, not known to the authors
**16. Description of sample**	What are the important characteristics of the sample? *e.g. demographic data, date*	All participants were members of ad hoc expert committees installed to advice on matters concerning the COVID-19 pandemic in Germany
**Data collection**		
**17. Interview guide**	Were questions, prompts, guides provided by the authors? Was it pilot tested?	Interview guide was not pilot tested, and participants did not get the questions prior to the interview. However the interview guide included open questions and prompts, that were adapted iteratively
**18. Repeated Interviews**	Were repeat interviews carried out? If yes, how many?	No
**19. Audio/visual recording**	Did the research use audio or visual recording to collect the data?	Audio recording
**20. Field notes**	Were field notes made during and/or after the interview or focus group?	Yes, both during and after the interviews
**21. Duration**	What was the duration of the interviews or focus group?	Lasted between 28 and 57 minutes with an average of 48 minutes
**22. Data Saturation**	Was data saturation discussed?	No
**23. Transcripts returned**	Were transcripts returned to participants for comment and/or correction?	No
**Domain 3: analysis and findings**		
**Data analysis**		
**24. Number of data coders**	How many data coders coded the data?	Two
**25. Description of the coding tree**	Did authors provide a description of the coding tree?	Yes in dedicated Appendix 3

**26. Derivation of themes**	Were themes identified in advance or derived from the data?	derived from the data
**27. Software**	What software, if applicable, was used to manage the data?	MAXQDA
**28 Participant checking**	Did participants provide feedback on the findings?	No
**Reporting**		
**29. Quotations presented**	Were participant quotations presented to illustrate the themes / findings? Was each quotation identified? e.*g. participant number*	Yes, quotations were presented. However, they were only attributed to the pseudonyms of the participants to safeguard the participants' anonymity
**30. Data and findings consistent**	Was there consistency between the data presented and the findings?	Yes
**31. Clarity of major themes**	Were major themes clearly presented in the findings?	1)Structure of advisory process 2) expert deliberation within the advisory process 3) Reflections on the advisory process
**32. Clarity of minor themes**	Is there a description of diverse cases or discussion of minor themes?	Yes, within the examples presented of the individual major themes' sections
