# Supplementary material for: "There Is No One Truth": A Qualitative Analysis of Advisory Committees in COVID-19 Policy-Making in Germany
Source: Int J Health Policy Manag. 2026 Aug 1;15:9424. doi: 10.34172/ijhpm.9424 (PMC13770243; doi:10.34172/ijhpm.9424)
Supplement: Supplementary file 2 [file ijhpm-15-9424-s002.pdf]

Article title: "There Is No One Truth": A Qualitative Analysis of Advisory Committees in COVID-19 Policy-Making in Germany

Journal name: International Journal of Health Policy and Management (IJHPM)

Authors' information: Elisa Braun[1,2,3]¶, Kerstin Sell[1,2]¶, Evelien de Hoop[3], Karin Geffert[1,2]*

[1]Institute for Medical Information Processing, Biometry and Epidemiology, LMU Munich, Munich, Germany.

[2]Pettenkofer School of Public Health, Munich, Germany.

[3]Athena Institute, Science Faculty, Vrije Universiteit Amsterdam, The Netherlands.

***Correspondence to:** Karin Geffert; Email: kgeffert@ibe.uni-muenchen.de

Citation: Braun E, Sell K, de Hoop E, Geffert K. "There is no one truth": a qualitative analysis of advisory committees in COVID-19 policy-making in Germany. Int J Health Policy Manag. 2026;15:9424. doi:10.34172/ijhpm.9424

Supplementary file 2. Interview Guide

#	Min	Open question	Deepening question	Prompt	Aim
**Introduction**					
**0**	3	- Name<br>- Role in the committee<br>- Discipline represented in the committee<br>- Who invited you to become part of the committee?<br>- In your opinion, why were you invited to the panel?<br>- Have you been advising policymakers in another context?			Introduction
		In the further course of the interview, we would like to focus mainly on your activities in the expert committee(s) in [federal state]			
**Practices of the Committee**					
**Could you start by telling us something about the committee and its background and tasks?**					Characterizing Practices of the committee

**Committee Meeting**					
**1**	10	How did the committee operate?	Were there any occasions for consultation?	Convening of a meeting by policymakers; events in the pandemic context; regular intervals independent of this	
				Did they have to agree on a joint recommendation, was it about discussing different points of view or something else?	
**2**		What was the objective / task of the committee?	What were the intended end products/outputs of a meeting?	In Thuringia, there were committee meetings at which either specific questions were answered or recommendations were drawn up on specific topics. What did this look like in your committee?	
				Primary data, expert opinions, concrete proposals	
		Who was present at the committee meeting?			
**Expert Deliberation**					
**Could you tell us a bit more about how the panel works, especially with regard to the use of expertise and scientific knowledge?**					
**3**	15	What did a meeting look like? How did it go? How did you arrive at the desired end product?	How did the expert discussions look like?		Characterisation of the deliberation of expertise and evidence
			**What was your experience of the use of evidence and expertise? How did you experience the availability of relevant information in your discipline?**	Lived expertise / personal experience; studies (what kind of studies?); anecdotal evidence; assessment, interpretation of the current data situation; recommendations from other countries; other experts' opinions	

			On what basis were the experts' statements made?	if politicians were present: what was their role? how did they give input?	
			How did they perceive the scientific discourse between representatives of different disciplines?	How was it ensured that presentations from one specialised field were understandable for all those present?	Assessment of interdisciplinary competences
			How were different assessments of the situation dealt with?	In Thuringia, the level of consensus or disagreement was indicated in your letters. How was this handled in your committee?	Characterisation of the deliberation of expertise and evidence
			How did scientific uncertainty influence the panel's work? How was scientific uncertainty dealt with? How was it taken into account and communicated in the further consultation process?	In Thuringia, the data situation on the respective topics was stated in the letters of recommendation. How was this handled in your committee?	
			How was the political context taken into account?	Especially for proposals for action: Weighing up the options? Several options to choose from or 1 concrete proposal? Checking the feasibility? Language?	
			How would you rate the commitment or involvement of the representatives of the different disciplines? Do you feel that equal attention was paid to all disciplines?		
**Communicating the results of the counselling processes**					
**Could you explain to me in more detail how the results of the committee were communicated?**					

**4**	5	In what form and to whom (which target group) were the results of the consultations passed on?	In Thuringia, the results of the committee meetings were published on the state government's website and were therefore accessible not only to policymakers but to all interested citizens. [Thuringia: Are you aware of any reasons for this?]	Meetings, letters, writing	Explanation of appropriate communication strategies
			Were there reasons for the chosen form of presentation?	Comprehensibility for policymakers who are not experts in the field (scientific expertise)	
**End**					
**[If you have been on several committees: Where do you see the main differences in the way the committees work?]**					
**5**	10	Would you like to add something or emphasise something again?			
