# Supplementary material for: "There Is No One Truth": A Qualitative Analysis of Advisory Committees in COVID-19 Policy-Making in Germany
Source: Int J Health Policy Manag. 2026 Aug 1;15:9424. doi: 10.34172/ijhpm.9424 (PMC13770243; doi:10.34172/ijhpm.9424)
Supplement: Supplementary file 3 [file ijhpm-15-9424-s003.pdf]

Article title: "There Is No One Truth": A Qualitative Analysis of Advisory Committees in COVID-19 Policy-Making in Germany

Journal name: International Journal of Health Policy and Management (IJHPM)

Authors' information: Elisa Braun[1,2,3]¶, Kerstin Sell[1,2]¶, Evelien de Hoop[3], Karin Geffert[1,2]*

[1]Institute for Medical Information Processing, Biometry and Epidemiology, LMU Munich, Munich, Germany.

[2]Pettenkofer School of Public Health, Munich, Germany.

[3]Athena Institute, Science Faculty, Vrije Universiteit Amsterdam, The Netherlands.

***Correspondence to:** Karin Geffert; Email: kgeffert@ibe.uni-muenchen.de

Citation: Braun E, Sell K, de Hoop E, Geffert K. "There is no one truth": a qualitative analysis of advisory committees in COVID-19 policy-making in Germany. Int J Health Policy Manag. 2026;15:9424. doi:10.34172/ijhpm.9424

Supplementary file 3. Code Book

**Mode of operation of the committee**			
	Procedure of a discussion round		
	Intended end products		
		Process to get to the end product	
	General rational for the committee		
		Rational for the committee composition	
		Rational for the invitation to the committee	
	Consulting		
	Fashion of consultation		
		Attendees of meetings	
			Influence of attendees on the deliberation
	Objective of the committee		
	Consultation scheme and counselling occasions		
	Self-establishment of the committee, finding work processes, and to each other		
	Session topics, how they were structured and who proposed them		
**Comments on the committee**			
	Secrets/ Transparency		
	negative		

positive
**Culture of deliberation**
Foundations of the advice production
Analogies
Observations
Use of evidence
Data
Literature
Studies
Use of expertise
Experience
Expert opinion
Use of logic
Input from external experts
Rationality
Personal bias
Joint Sense making of evidence
Scientific uncertainty
Data availability
**Interaction with politicians**
Relationship with politicians and the form of exchange with politicians
Committee as a political instrument
Political agenda
Input of the politicians during a session
**Communication of the committee**
Recipients of advice, counseling end products
Justification of the chosen form of communication
Form of communication
**Context**
Best practice
Learnings

	Limitations
	Pandemic development
	Political circumstances
	Accessibility of data
**Permanent committees**	
	Mode of operation
	End product
	Foundations of the advice production
**Tailoring Evidence**	
	Feasibility of implementing scientific advice
**Perception of interdisciplinarity**	
	Relationship between the experts on a panel
	Discipline of the interview participants
	Hurdles of interdisciplinary/large committees
	Multi-disciplinary work
	Prioritisation of disciplines
	Communication between the disciplines
	Appreciation of the disciplines
**Area of responsibility (self-perception)**	
	Advisory process during the pandemic
	Influence on political decisions
	Perception of the advisory process
