# Supplementary material for: "There Is No One Truth": A Qualitative Analysis of Advisory Committees in COVID-19 Policy-Making in Germany
Source: Int J Health Policy Manag. 2026 Aug 1;15:9424. doi: 10.34172/ijhpm.9424 (PMC13770243; doi:10.34172/ijhpm.9424)
Supplement: Supplementary file 5 [file ijhpm-15-9424-s005.pdf]

Article title: "There Is No One Truth": A Qualitative Analysis of Advisory Committees in COVID-19 Policy-Making in Germany

Journal name: International Journal of Health Policy and Management (IJHPM)

Authors' information: Elisa Braun[1,2,3]¶, Kerstin Sell[1,2]¶, Evelien de Hoop[3], Karin Geffert[1,2]*

[1]Institute for Medical Information Processing, Biometry and Epidemiology, LMU Munich, Munich, Germany.

[2]Pettenkofer School of Public Health, Munich, Germany.

[3]Athena Institute, Science Faculty, Vrije Universiteit Amsterdam, The Netherlands.

***Correspondence to:** Karin Geffert; Email: kgeffert@ibe.uni-muenchen.de

Citation: Braun E, Sell K, de Hoop E, Geffert K. "There is no one truth": a qualitative analysis of advisory committees in COVID-19 policy-making in Germany. Int J Health Policy Manag. 2026;15:9424. doi:10.34172/ijhpm.9424

Supplementary file 5. Recruitment Process

Region	Members	Eligible members	Members with an e-mail	Contacted Members	Invited Members	Participant Number
Bund	18	18	16	15	3	P02-Bund, P08-Bund, P10-Bund
BY	14	13	11	10	1	P03-BY
NRW	12	11	10	10	0	n.d.
RP	7	6	5	5	0	n.d.
SH	10	8	8	8	2	P01-SH, P12-SH
TH	13	13	12	12	5	P04-TH, P05-TH, P06-TH, P07-TH, P11-TH
**Total**	80	75	68	66	12	
